# Pattern Recognition Receptors and the Host Cell Death Molecular Machinery

**DOI:** 10.3389/fimmu.2018.02379

**Published:** 2018-10-16

**Authors:** Gustavo P. Amarante-Mendes, Sandy Adjemian, Laura Migliari Branco, Larissa C. Zanetti, Ricardo Weinlich, Karina R. Bortoluci

**Affiliations:** ^1^Departamento de Imunologia, Instituto de Ciências Biomédicas, Universidade de São Paulo, São Paulo, Brazil; ^2^Instituto de Investigação em Imunologia, Instituto Nacional de Ciência e Tecnologia (INCT), São Paulo, Brazil; ^3^Molecular Signaling and Cell Death Unit, Inflammation Research Center, VIB, Ghent, Belgium; ^4^Department of Biomedical Molecular Biology, Ghent University, Ghent, Belgium; ^5^Departamento de Ciências Biológicas, Universidade Federal de São Paulo, Diadema, Brazil; ^6^Centro de Terapia Celular e Molecular (CTC-Mol), Universidade Federal de São Paulo, São Paulo, Brazil; ^7^Instituto Israelita de Ensino e Pesquisa, Hospital Israelita Albert Einstein, São Paulo, Brazil

**Keywords:** PRR, pathogen recognition receptor, apoptosis, necroptosis, pyroptosis, inflammation

## Abstract

Pattern Recognition Receptors (PRRs) are proteins capable of recognizing molecules frequently found in pathogens (the so-called Pathogen-Associated Molecular Patterns—PAMPs), or molecules released by damaged cells (the Damage-Associated Molecular Patterns—DAMPs). They emerged phylogenetically prior to the appearance of the adaptive immunity and, therefore, are considered part of the innate immune system. Signals derived from the engagement of PRRs on the immune cells activate microbicidal and pro-inflammatory responses required to eliminate or, at least, to contain infectious agents. Molecularly controlled forms of cell death are also part of a very ancestral mechanism involved in key aspects of the physiology of multicellular organism, including the elimination of unwanted, damaged or infected cells. Interestingly, each form of cell death has its particular effect on inflammation and on the development of innate and adaptive immune responses. In this review article, we discuss some aspects of the molecular interplay between the cell death machinery and signals initiated by the activation of PRRs by PAMPs and DAMPs.

## Introduction

In 1989, Charles Janeway Jr. proposed the existence of a collection of receptors expressed by innate immune cells responsible for detecting conserved products of microbial origin (1). After 25 years of intense research, fierce debates, and a Nobel Prize granted on this subject, it is unquestionable that Janeway's ingenious idea has revolutionized our understanding of the immune system. Indeed, his seminal article is considered as one of the pillars of immunology (2).

The so-called Pattern Recognition Receptors (PRRs) are proteins capable of recognizing molecules frequently associated with pathogens (aka Pathogen-Associated Molecular Patterns—PAMPs). A more comprehensive description of PRRs and their signaling transduction pathways can be found elsewhere (3). Briefly, PRRs can be found associated to subcellular compartments, such as the cellular and endosomal membranes, the cytosol, as well as extracellularly, in secreted forms present in the bloodstream and interstitial fluids (3). There are four major sub-families of PRRs—the Toll-like receptors (TLRs), the nucleotide-binding oligomerization domain (NOD)- Leucin Rich Repeats (LRR)-containing receptors (NLR), the retinoic acid-inducible gene 1 (RIG-1) -like receptors (RLR; aka RIG-1-like helicases—RLH), and the C-type lectin receptors (CLRs) (4). As predicted by Janeway, the engagement of PRRs on the innate immune cells induces co-stimulatory signals for the adaptive immune cells (particularly T lymphocytes) (5). In addition, they activate microbicidal and pro-inflammatory responses required to eliminate (or at least to contain) infectious agents, including the induction of infected cell death (6), as discussed below.

Another ingenious idea came from Polly Matzinger (7), who proposed that the immune system is less concerned with the origin of the antigens (self vs. non-self) than with the context of their encounter with our body (tissue damage vs. tissue homeostasis). In her “Danger Theory,” Matzinger suggested that during tissue stress or damage, endogenous molecules are released or activated and initiate or propagate the inflammatory response, which, among other things, empower antigen-presenting cells to activate the adaptive immune response. Today, these molecules are collectively known as DAMPs (Damage-Associated Molecular Patterns). Importantly, soon enough it became clear that similarly to PAMPs, DAMPs could also engage PRRs.

These two theories together put forward the idea that our body is equipped to distinguish “healthy,” homeostatic tissue turnover or encounters with foreign “friendly” microorganisms, from potential “danger” that may come from pathogens and/or tissue damage.

## Cell death programs

Molecularly controlled forms of cell death are part of a very ancestral mechanism involved in key aspects of the physiology of multicellular organism, including the elimination of unwanted, damaged or infected cells. Importantly to our discussion, cell death can have a direct or an indirect impact upon the course of infection, as the elimination of infected cells may eradicate or at least restrain the growth of a given pathogen. Moreover, the recognition of dying cells or their by-products modulates both inflammatory and immune responses. In the following sections, we will briefly describe the mechanisms that govern the three major types of molecularly controlled forms of cell death, namely apoptosis, necroptosis and pyroptosis, that participate in host defense through elimination of infected cells, and how they are regulated by signals derived from PRRs. For information regarding other cell death modes please refer to the work published by the Nomenclature on Cell Death Committee 2018 (8)

### Apoptosis

Apoptosis was the first type of programmed cell death to be described, initially based on morphological features that distinguished it from necrosis, an uncontrolled, accidental form of cell death observed upon extreme physicochemical insults (9). In this regard, apoptosis is characterized by chromatin condensation, nuclear fragmentation, cell shrinkage with formation of cellular membrane blebs, and, finally, cellular disintegration into fragments known as apoptotic bodies (10). Importantly, during apoptosis, the plasma membrane integrity is preserved, avoiding the release of intracellular contents to the extracellular milieu. This feature contributes to the concept that apoptosis is an (relatively) inflammatory-silent form of cell death. Indeed, recognition and elimination of apoptotic cells during physiological circumstances, such as tissue/organ sculpture during development and tissue homeostasis, occurs without the cardinal signs of inflammation. In addition, it is well established that recognition of apoptotic cells by macrophages, in particular, results in the production of anti-inflammatory molecules, such as TGF-β and PGE_2_ (11). On the other hand, it is also known that apoptotic cells release a series of so-called “find-me” signals, such as extracellular ATP and lysophosphatidylcholine (LPC), capable of recruiting phagocytes to the site of apoptotic corpses, characterizing, therefore, at least one aspect of an inflammatory reaction (12, 13). Besides, more recently, it was shown that apoptosis initiated via the FAS/CD95 death receptor is associated with the release of chemokines and other immunologically active proteins that coordinates the migration of phagocytes and proper removal of apoptotic cells (14). Taken together, it is reasonable to say that although not completely “silent,” apoptosis is a form of cell death that does not trigger an overt inflammatory response.

From the molecular point of view, much of our knowledge about the regulation of apoptosis came from works with the nematode *Caenorhabditis elegans*. In a series of elegant studies, Bob Horvitz and colleagues identified four crucial genes (*Ced-3, Ced-4, Ced-9, and Egl-1*) responsible for the control of developmental cell death in *C. elegans* (15), which granted him the Nobel Prize in Physiology or Medicine in 2002, together with John Sulston and Sidney Brenner, “*for their discoveries concerning genetic regulation of organ development and programmed cell death*.” Soon after Horvitz discoveries, it became clear that cell death in *C. elegans* and apoptosis in mammals shared a very similar, phylogenetically conserved mechanism. Apoptosis is executed by certain members of a family of cysteine aspartate-specific proteases called caspases (16–18). Importantly, not all caspases induces apoptosis. Caspases-1, -4, -5, -11, -12, -13, and-14 are inflammatory caspases not related to the initiation or execution of the apoptotic program. Caspases are produced as an inactive pro-form (zymogen) that can be activated either through proteolytic processing by upstream caspases (in the case of caspases-3, -6, and-7) or via dimerization in the context of multimolecular platforms, such as the apoptosome (caspase-9), the DISC (death-inducing signaling complex) (caspases-8 and-10), the PIDDosome (caspase-2), and the inflammasome (caspase-1 and-11) (16). Executioner or effector caspases, such as caspase-3, -6, and-7 (and CED-3 in *C. elegans*), are responsible for the induction of the morphological as well as the biochemical features associated with apoptosis, including oligonucleosomal DNA fragmentation and externalization of phosphatidylserine (PS) residues from the inner to the outer leaflet of the plasma membrane (19). Interestingly, in mammals, although the inhibition of effector caspases prevents apoptosis, it does not preclude cell death, which proceeds with different morphological and biochemical characteristics (20). Because of this, it has been proposed that apoptosis in mammals may not be actually a cell death mechanism, but perhaps a termination step of a cell-death program aimed to properly dispose damaged or unwanted cells without initiating inflammatory responses (18).

**Figure 1:**
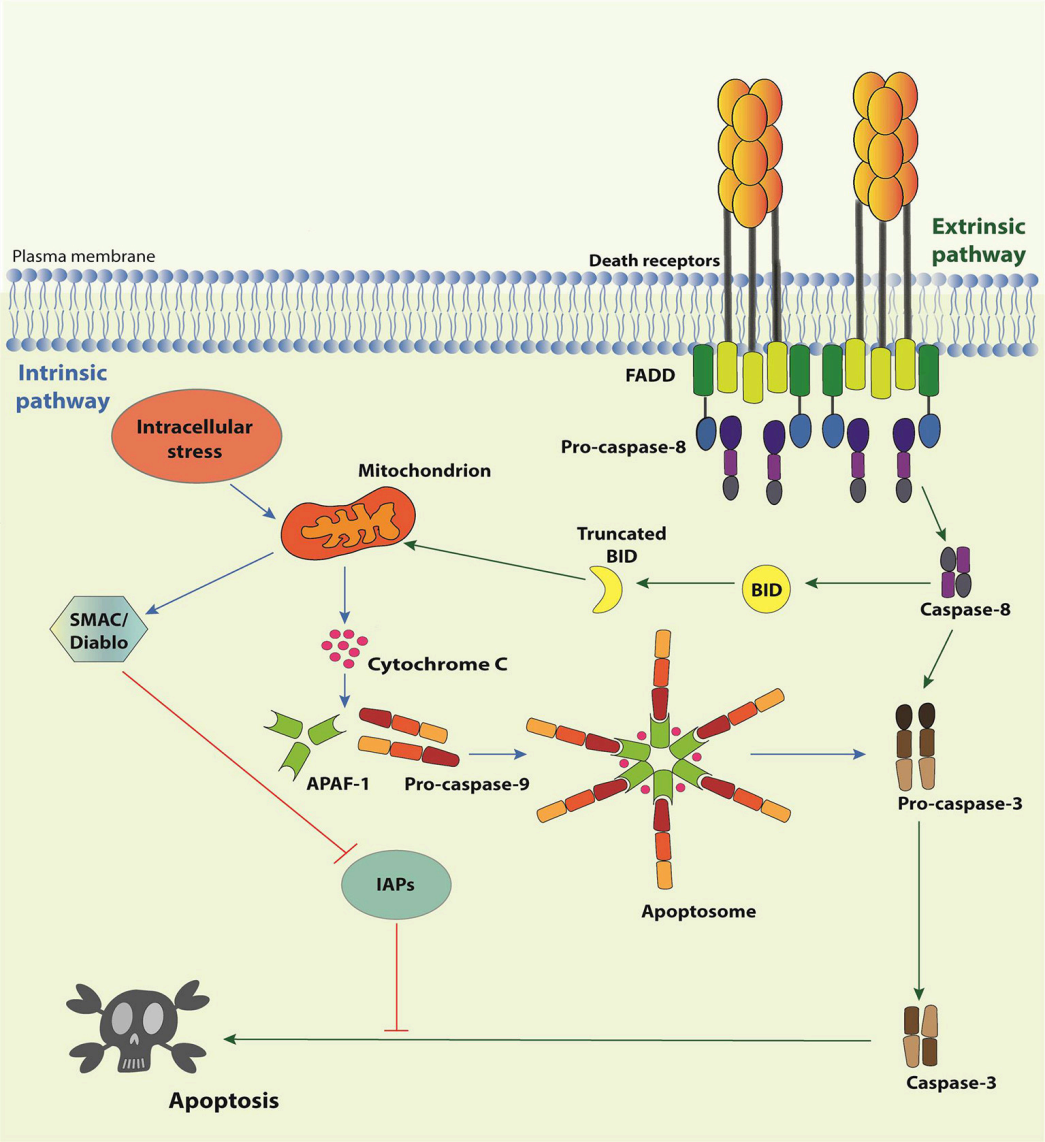
Apoptosis pathways. The Intrinsic Pathway of apoptosis is activated when intracellular “stresses,” such as DNA or cytoskeleton damage or absence of growth/survival factors, are “perceived” by BH3-only members of the Bcl-2 family. These molecules become activated and migrate to the mitochondria where they facilitate or actively induce the release of apoptogenic factors, such as cytochrome c and SMAC/Diablo, to the cytosol. Cytochrome c associates with APAF-1 and pro-caspase-9 to form the apoptosome, resulting in the activation of caspase-9, which activates the effector caspases-3, -6, and -7, responsible for the biochemical and morphological modifications associated to apoptosis. SMAC/Diablo participates by preventing inhibition of caspases by IAPs. The extrinsic pathway of apoptosis initiates by the engagement of Death Receptor by their cognate Death Receptor Ligands causing the formation of the Death-inducing signaling complex (DISC). DISC is formed by the intracellular portion of the Death Receptors, the adaptor proteins TRADD and/or FADD and the pro-caspase-8 (or pro-caspase-10). Activated caspase-8 may directly activate the effector caspases or process the BH3-only protein Bid. Truncated Bid migrates to mitochondria and activates the extrinsic pathway of apoptosis.

There are two signaling pathways of apoptosis (Figure 1). The intrinsic pathway deals with signals derived from intracellular stress, such as DNA damage, oxidative stress, dysregulation of Ca^2+^ homeostasis, interference with the cytoskeleton structure, endoplasmic reticulum stress, etc. Its first layer of regulation comprises the differential expression/activation of BCL-2 family members, responsible for controlling the mitochondria outer membrane permeabilization (MOMP) (21). When the pro-apoptotic stress is too strong for a given cell, MOMP allows the selective release of certain mitochondrial proteins, such as SMAC (second mitochondria-derived activator of caspases)/Diablo (direct IAP binding protein with low pI), HtrA2 (high temperature requirement protein A2)/Omi, and cytochrome c to the cytosol. Cytochrome c associates with APAF-1 (apoptosis-activating factor-1), the mammalian CED-4 homolog, and pro-caspase-9, thereby assembling the apoptosome and enabling caspase-9 to activate the downstream effector caspases. SMAC/Diablo and HtrA2/Omi facilitate apoptosis by preventing the inhibitory action of the inhibitors of apoptosis proteins (IAPs) on the effector caspases. The extrinsic pathway, in comparison, is initiated by the interaction of trimeric, extracellular ligands (TNF-α, CD95L, and TRAIL) to their cognate receptors (TNFR1, CD95 and TRAILRI, or TRAILRII, respectively) present on the plasma membrane (10, 22, 23). The stimulation of these so-called death receptors (DRs) leads to the recruitment of adaptor molecules, such as TRADD (Tumor necrosis factor receptor type 1-associated death domain protein) and/or FADD (Fas-associated protein with death domain), and the pro-caspase-8, giving rise to the conventional DISC. Next, caspase-8 directly activates the effector caspases or amplifies the cell death signal by engaging BID (BH3 interacting-domain death agonist), a pro-apoptotic member of the BCL-2 (B-cell lymphoma 2) family, leading to MOMP, cytochrome c release and assembly of the apoptosome (Figure 1). It is important to mention that the activation of caspase-8 in the context of DISC can be regulated by c-FLIP (cellular FLICE-like inhibitory protein), a catalytically-dead caspase-8 homolog (24).

In some instances, apoptosis can also be triggered by TLR stimulation, as a defense mechanism against infection. TLR2 was the first PRR to be associated with induction of apoptosis, by virtue of its ability to recruit FADD via MyD88 (Myeloid differentiation primary response 88), and the consequent activation of caspase-8 (25). Likewise, bacterial lipoproteins were reported to trigger apoptosis through this TLR2 pathway (26, 27) and *Mycobacterium tuberculosis* was also shown to induce TLR-2/caspase-8-dependent apoptosis in macrophages (28). Interestingly, TLR3-induced apoptosis is mediated via TRIF (TIR-domain-containing adapter-inducing interferon-β), which interacts with RIPK1 (Receptor Interacting Serine/Threonine Kinase 1) through its RHIM (RIP homotypic interaction motif) domain (please refer to necroptosis section for further information on these protein-protein interactions). FADD is then recruited, and activates caspase-8 leading to apoptosis (25, 29). In human keratinocytes, poly I:C-induced apoptosis required the stimulation of TLR3 and its adaptor TRIF, thus inducing caspase-8 activation (30); the same molecules were shown to induce apoptosis in human breast cancer cells (31). Not surprisingly, TLR4 can induce apoptosis either via MyD88 or TRIF, and depending on the cell type or conditions engage the extrinsic or intrinsic pathways. For instance, *Yersinia* was shown to induce TLR4-mediated apoptosis of macrophages through TRIF (32, 33). TRIF-mediated apoptosis seems to be executed through the extrinsic pathway, with no evidence of the involvement of the mitochondrial pathway (34). Interestingly, UV irradiation was shown to induce apoptosis in murine macrophages through TLR4 and MyD88 (35). Despite these observations and a number of other examples that we have not presented here, it is important to emphasize that PRR-induced apoptosis is a relatively minor event compared to all other triggers of apoptosis and that PRR activation leads preferentially to other forms of regulated cell death, as we will discuss below.

### Necroptosis

Evidence of a molecularly controlled necrotic cell death was first provided by studies showing that Tumor Necrosis Factor Receptor 1 (TNFR1) and CD95 ligation were capable of inducing necrosis, particularly when caspase activity was inhibited (36, 37). This idea was further supported by a study that demonstrated that the cowpox virus could induce necrosis in porcine kidney cells when it harbored the caspase inhibitor CrmA (cytokine response modifier A) (38). This cell death mode was named “Necroptosis,” as it reflects the existence of a molecular pathway (like apoptosis) but with a necrotic phenotype.

**Figure 2:**
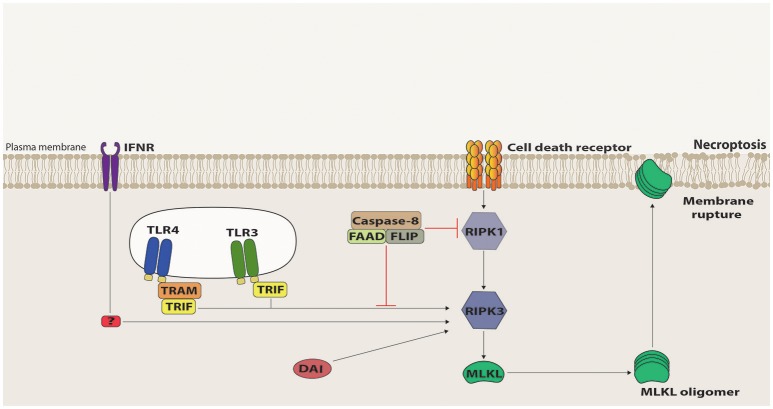
Necroptosis signaling. Death Receptor (DR)-induced necroptosis requires RIPK1 kinase activity to recruit RIPK3 that, in turn, recruits and activates MLKL via phosphorylation of its pseudokinase domain. Once phosphorylated, MLKL oligomerizes and migrates to the plasma membrane, where it interacts with phosphatidylinositol phosphates and induces membrane destabilization and rupture. Necroptosis signaling mediated by TRIF, IFNR, and DAI can directly activate RIPK3 and, in this case, RIPK1 acts as a negative regulator, mostly by recruiting to the signaling platform the suppressive complex containing Caspase-8, FADD and c-FLIP.

The first molecule to be identified in the necroptotic pathway was RIPK1 as its kinase activity inhibitor, necrostatin-1 (Nec-1), was shown to suppress cell death triggered by caspase inhibition during TNFR1/Fas stimulation (39). RIPK1 has been previously involved in apoptotic and survival pathways, functioning as a scaffold protein to the assembly of the respective signaling platforms (40). Contrastingly, the RIPK1 kinase activity is indispensible for death receptor-triggered necroptosis, as its auto-phosphorylation induces a conformational change that allows RIPK1 to recruit, via their respective RHIM domains, the next member of this pathway, namely RIPK3 (41–43). Once recruited, RIPK3 gets activated by auto-phosphorylation and forms an amyloid-like structure, which promotes the recruitment and activation of Mixed Lineage Kinase Domain-Like (MLKL) (42, 44–47). RIPK3-phosphorylated MLKL oligomerizes and translocates to the plasma membrane, where it interacts with phosphatidylinositides and induces plasma membrane disruption [(48–51); Figure 2). Distinct effector mechanisms were raised to account for the MLKL-driven permeabilization of the plasma membrane, either directly by pore or cation channel formation, or indirectly, by activation of TRPM or other ion channels (48–52). It is still unclear, however, which of these mechanisms are physiologically relevant. Nonetheless, in all cases, MLKL induces a loss of osmolality control, which causes cell swelling and membrane rupture. Recently, ESCRT-III machinery was suggested to counter these effects by shedding out the MLKL-damaged plasma membrane regions (53).

Necroptosis can be initiated by a variety of signals. The first to be described and most thoroughly studied was TNFR1 ligation (36). Upon its ligation, TNFR1 typically assembles a multimolecular complex (Complex I) composed by TRADD, RIPK1, TRAF2, TRAF5, cIAP1, cIAP2, and LUBAC (linear ubiquitin chain assembly complex), which is involved in NF-κB activation, pro-inflammatory cytokines synthesis and cell survival (54). Sustained TNFRI ligation leads to CYLD-mediated deubiquitination of this complex, which disassembles, allowing the formation of a secondary complex (Complex II) in the cytosol, constituted by TRADD, FADD, RIPK1, caspase-8, and occasionally c-FLIP (54). As pointed out above, when c-FLIP levels are low, caspase-8 forms active homodimers and triggers downstream events that culminate in apoptosis. However, in the absence of FADD, c-FLIP or a functional caspase-8, TNFRI signaling results in the recruitment of TRADD and RIPK1, forming a platform called complex IIb or necrosome, wherein RIPK3 and MLKL are activated to execute necroptosis (55). Although slightly differing on how RIPK1 is brought to the complex, this molecule has also a central role in Fas and TRAILR-induced necroptosis, as RIPK1 is, in all these cases, mandatory to recruit RIPK3 via their RHIM homotypic domain interactions (54).

Necroptosis can also be triggered by PRRs, such as TLR3 and TLR4, intracellular sensing proteins, such as DAI, RIG-I and MDA-5 as well as interferon signaling [(56–59); Figure 2). Intriguingly, however, RIPK1 is dispensable for or even inhibitory of the necrosome formation during TLR3-, TLR4-, DAI-, and interferon-mediated necroptosis (57, 60). In these cases, RIPK3 is directly recruited to the signaling platforms, and the presence of RIPK1 slows down or halts the RIPK3-mediated activation of MLKL (60, 61). The ability of RIPK1 to recruit FADD, and consequently, caspase-8 and FLIP accounts, at least in part, for its inhibitory property. Therefore, from the molecular point of view, necroptosis ought to be defined as a RIPK3-dependent form of cell death.

Many other stimuli have been described as capable to induce necroptosis, ranging from UV irradiation, chemotherapeutic drugs (such as cisplatin, etoposide, and staurosporine), natural compounds (such as shikonin and its analogs), to DNA damage, hypoxia, ischemia/reperfusion and oxidative stress (62). The signaling pathways that lead to necroptosis in each of these cases are still to be fully elucidated. Further studies are required to evaluate whether they are dependent on RIPK1 and also whether they directly signal to a RIPK3-activating platform or indirectly, via up regulation of a classic necroptotic inducer, such as TNF or FasL. For example, UV irradiation was reported to induce necroptosis via TNF upregulation but also via spontaneous aggregation of RIPK1 and RIPK3, independently of any death receptor ligation (29, 63). Particularly puzzling is the fact that shikonin, a naphthoquinone compound obtained from a plant extract, can induce necroptosis even in the absence of FADD/caspase-8/FLIP inhibition, which is thought to be mandatory for this type of cell death (64). Thus, either this compound can itself somehow block their activity, or it shall be instrumental to decipher alternative ways in which MLKL is activated and necroptosis is executed.

Nonetheless, despite the different mechanisms that initiate necroptosis, in all cases cells undergo rapid MLKL-mediated plasma membrane permeabilization with consequent release of intracellular contents, including many DAMPs, such as lysosomal proteases, DNA, mtDNA, ATP, and HMGB1 [(55); Table 1). Therefore, similarly to pyroptosis (see below), necroptosis is considered a pro-inflammatory form of cell death. Even so, it is still to be determined whether the pro-inflammatory properties of necroptotic cells are the result of the intracellular content leakage or, rather, they can actively produce and/or modify specific DAMPs. Evidence for the latter comes from ESCRT-III-deficient cells that undergo necroptosis much faster, which limits the amount of inflammatory cytokines and chemokines produced and hinders antigen cross-presentation (53). Moreover, both RIPK3 and MLKL have been associated with inflammasome and NF-κB activation, supporting the notion that the pro-inflammatory potential of necroptotic cells goes beyond the passive release of their intracellular content (110–112).

**Table 1:** DAMPs released by cell death and its role in the immune system.

**DAMPs**	**Immunogenic function**	**Receptors**	**Related cell death**	**References**
Adenosine triphosphate (ATP)	DC and Mφ activation Inflammasome activation	P2Y2,6,12, P2X1,3,7 NLRP3	Apoptosis Pyroptosis Necroptosis NCD	(65–69)
Annexin A1 (ANXA1)	“Eat me” signal Immunogenicity	FPR1	Apoptosis	(70)
ASC specks	Lysosomal damage IL-1β activation	unknown	Pyroptosis	(71)
Calreticulin	“Eat me signal” Immunogenicity	CD91	Apoptosis	(72, 73)
Cyclophilin A	Cytokine induction	CD147	Necroptosis NCD	(74, 75)
Defensin α	Antimicrobial Anti-inflammatory	CCR2, CCR6, TLR4	Apoptosis NCD	(76)
Heat shock proteins (HSPs)	Monocytes and neutrophils attraction DC maturation	CD91, TLR2, TLR4, SREC1 and FEEL1	Necroptosis NCD	(77, 78, 79)
HMGB1	DCs and Mφ activation Cytokine activation	CXCR4, RAGE, TLR2,4,9	Apoptosis Necroptosis Pyroptosis	(69, 80–84)
HMGN1	Leukocyte recruitment DC maturation	TLR4	Necroptosis NCD	(85–87)
IL-1α	DC and Mφ activation Cytokine induction	IL-1R	Necroptosis Pyroptosis NCD	(88–90)
IL-33	Cytokine induction DC activation	ST2	Necroptosis NCD	(91, 92)
IL-6	Immune responses T cell differentiation	IL6R and GP130	Necroptosis NCD	(61, 93)
Lysophosphatidylcholine (LPC)	Monocyte and Mφ recruitment DC maturation “Eat me” signal	G2A	Apoptosis	(94, 95)
Mitochondrial DNA (mtDNA)	Mφ activation PMNs activation NLRP3 activation	TLR9	Necroptosis Pyroptosis	(96–99)
N-formyl peptides (NFP)	PMNs activation Monocyte activation	FPR1	NCD	(97, 100)
Nucleic acids (dsDNA/dsRNA)	DC activation Inflammasome activation Cytokine induction	TLR3, TLR7/8, TLR9, AIM2	Apoptosis Necroptosis Pyroptosis NCD	(68, 79, 101, 102)
Peroxiredoxin 1 (Prx1)	Cytokine induction CD maturation	TLR4	NCD	(103)
S100	Leukocyte recruitment Cytokine induction	RAGE, TLR4	Necroptosis NCD	(79, 104, 105)
SAP130	Mφ activation Neutrophil recruitment Cytokine induction	Mincle	Necroptosis NCD	(106, 107)
Uric acid	DC activation Inflammasome activation	P2X7, NLRP3	NCD	(108, 109)

*NCD stands for Necrotic Cell Death, which means the referred papers only characterized the cell death by its necrotic morphology. AIM2, absent in melanoma 2; ASC, Apoptosis-associated speck-like protein containing a CARD; CCR, CC chemokine receptor; CD, cluster of differentiation; CXCR, CXC chemokine receptor; DC, dendritic cells; FEEL-1, fasciclin EGF-like laminin-type EGF-like and link domain-containing scavenger receptor-1; FPR-1, formyl peptides receptor-1; G2A, G2 accumulation; GP130, Glycoprotein 130; HMGB1, high-mobility group box 1 protein; HMGN1, high-mobility group nucleosome-binding domain 1 protein; IL, interleukin; LRR and PYD domains-containing protein 3; Mincle, Macrophage inducible Ca^2+^-dependent lectin receptor; Mφ, macrophage; NLRP3, NACHT LRR and PYD domains-containing protein 3; P2XR, P2X receptor; P2YR, P2Y receptor; PMNs, polymorphonuclear leukocytes; RAGE, receptor for advanced glycation end-products; SAP130, Sin3A Associated Protein 130; SREC-I, Scavenger receptor expressed by endothelial cells; ST2, Interleukin 1 receptor-like 1; TLR, Toll-like receptor*.

Necroptotic cells not only induce a potent inflammatory response but they are also highly immunogenic, which may be instrumental against infection and during anti-tumoral responses. For example, mice injected with necroptotic cells present a higher CD8^+^ T cell cross-priming and increased tumor immunity when compared with animals injected with apoptotic cells (113, 114). Likewise, RIPK3 deficiency in mice inhibits immune cell infiltration and attenuates organ injury during sepsis (115). Therefore, given that necroptosis is highly immunogenic, disruption in the necroptotic pathway would be expected in some pathophysiological conditions. Indeed, it was reported that most of the *in vitro* transformed cells as well as human tumor samples have low or no expression of RIPK3 (116), and a cohort of chronic lymphocytic leukemia patients present down regulation of CYLD (117). Furthermore, patients with lower expression of RIPK3 or MLKL have worse prognosis for breast cancer or ovarian cancer, respectively (116, 118), suggesting that resistance to necroptosis is positively selected during tumor growth and/or development. This may be associated with an increased ability to evade immune attack, either by prolonging the lifespan of the transformed cells, by decreasing the availability of DAMPs, or by avoiding the activation of antigen-presenting cells during the immune responses. Therefore, induction of necroptosis in tumors may change its immunogenicity and promote a better immune response against it. This is particularly exciting, as we are currently witnessing novel and promising approaches in tumor treatment that are based on stimulation of the immune system. On the other hand, it is possible that the inflammation generated by necroptosis may promote tumor development by stimulating angiogenesis and metastasis (119). Therefore, thorough investigation of the benefits and pitfalls of inducing inflammatory cell death for each cancer type will be required in order to determine whether inducing necroptosis is indeed a good option in the specific cancer treatment.

Besides its impact on tumorigenesis and tumor progression, deficient necroptotic signaling can be detrimental during viral infection. Mice lacking RIPK3 are highly sensitive to vaccinia virus due to widespread infection (120). Likewise, RIPK3-deficient mice are more susceptible to Influenza A virus (IAV) than the wild-type animals (121). Remarkably, seasonal IAV, but not the 1918 and 2009 pandemic IAV strains, induces RIPK3-mediated immunogenic death of dendritic cells (122). The pandemic strains' ability to suppress necroptosis was mapped to the hemagglutinin (HA) genomic segment (122), indicating that either the pandemic strains' HA do not induce necroptosis or it may directly interfere with the necroptotic signaling pathway.

Keeping with the notion that suppressing necroptosis is advantageous to the infectious agent, there is accumulating evidence that viruses can encode molecules that are able to directly interfere with the necroptotic signaling. vIRA, a molecule expressed by MCMV that contains a RHIM-like domain blocks RIPK3 recruitment to RIPK1 and to DAI (57). MCMV expressing vIRA mutated in its RHIM domain produces an attenuated viremia in wild-type mice, which is reverted in RIPK3-deficient animals (57). Likewise, HSV-1 and HSV-2 express ICP-6 and ICP-10, respectively, which are able to suppress necroptosis in human cells through a similar RHIM-dependent mechanism (123, 124). Curiously, in mice, ICP-6 was shown to promote necroptosis through direct aggregation with RIPK3, restricting virus propagation (124, 125). A different mode of action was reported for the IE1-regulated gene product expressed by HCMV, which suppresses necroptosis downstream of RIPK3 activation and MLKL recruitment (126).

Bacteria can also induce necroptosis, at least *in vitro*. It is less clear, though, whether necroptosis plays a central role in bacterial infections *in vivo*. Loss of RIPK3 in combination with deletion or inhibition of caspase-8 or FADD renders mice susceptible to a number of pathogens, including *Yersinia* and *Citrobacter* (127, 128). However, the relative contribution of necroptosis and caspase-8-mediated apoptosis in these models were not yet tested, as caspase-8- or FADD–deficient animals are not viable (129–131).

Necroptosis, though, may not always be protective against infection. Macrophage death by necroptosis correlates with increased susceptibility to *Salmonella* infection (132). Also, HIV-specific CD8^+^ T cell response, which is a key indicator of infection control, is impaired due to increased necroptosis levels in this cell population (133). Taken together, necroptosis seems to be detrimental when it eliminates the population that is central for the immune control of the infection. In the other cases, necroptosis limits infection, mostly likely by destroying the pathogen's replicative niche through a cell death mode that generates a pro-inflammatory and immunogenic environment. However, it is important to note that, as mentioned above, RIPK3 and MLKL were shown to participate of additional signaling platforms, including inflammasome activation, NF-κB signaling and even apoptosis induction (134). Therefore, in the light of these novel RIPK3 and MLKL roles, it is essential to reevaluate the relative contribution of necroptosis to the phenotypes observed. A good illustration comes from the fact that while RIPK3-deficient mice are more susceptible to IAV, MLKL-deficient animals are not, indicating that necroptosis is not the sole RIPK3-mediated mechanism important in IAV control (121). In fact, it was shown that IAV also triggers RIPK3-mediated apoptosis, via recruitment of RIPK1, FADD and caspase-8. This was further supported by the fact that MLKL-caspase-8 double deficient mice present similar levels of susceptibility to IAV infection observed with the RIPK3-deficient animals (121). Another example is that RIPK3-deficient mice are less susceptible to *Staphylococcus aureus* lung damage and present reduced bacterial loads and inflammation, while MLKL-deficient animals present an opposite outcome, suggesting that these molecules have independent, non-necroptotic roles (135).

### Pyroptosis

**Figure 3:**
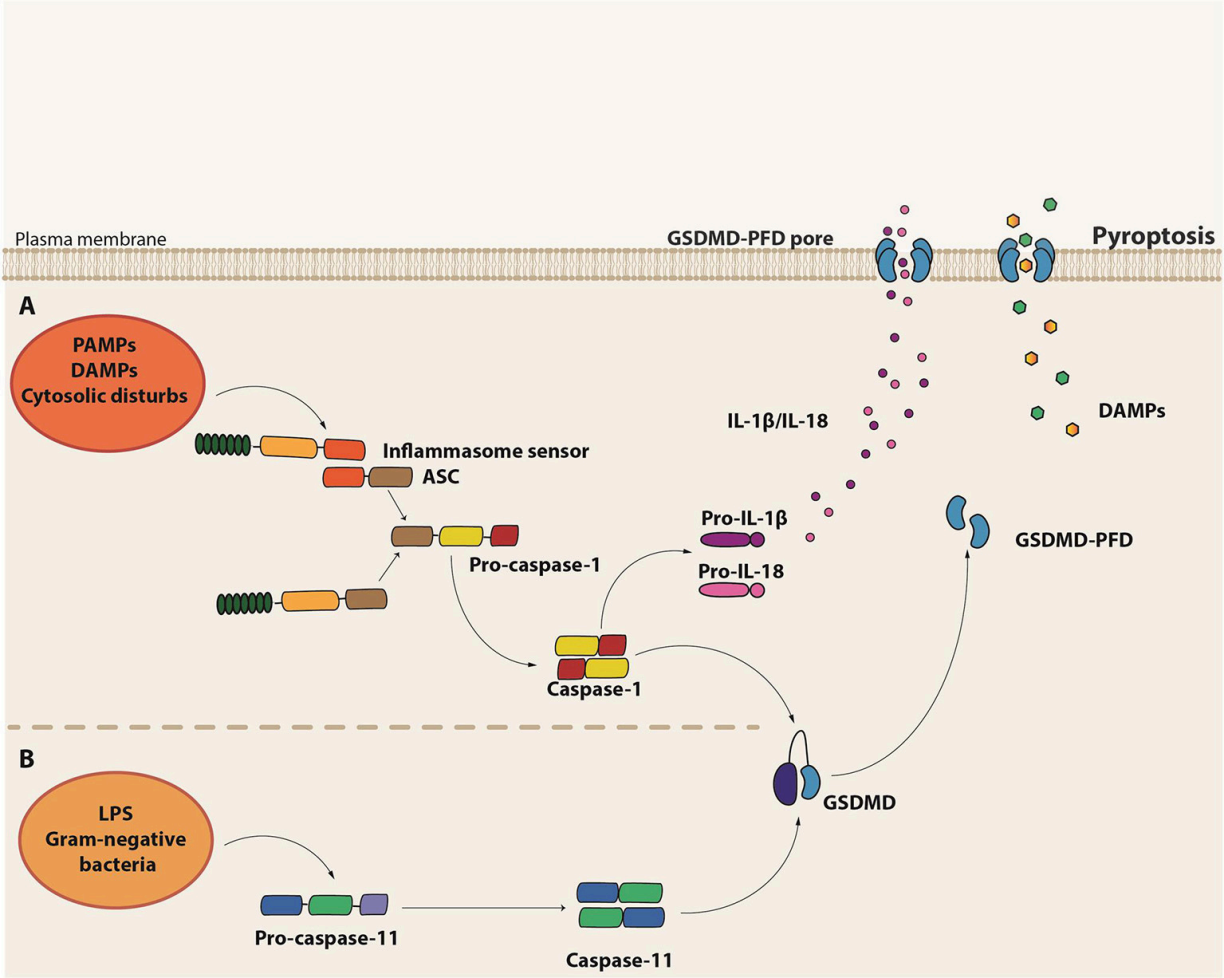
Molecular basis of pyroptosis. **(A)** Canonical inflammasome assembly upon sensing of PAMPs, DAMPs or other cytosolic disturbs leads to the recruitment and activation of caspase-1 directly or via the recruitment of the adaptor protein ASC. Caspase-1 induces the maturation of pro-IL-1β and pro-IL-18 into their active forms as well as cleavage of Gasdermin D (GSDMD). The GSDMD pore form domain (PFD) interacts with the plasma membrane to form the GSDMD pore, leading to the release of the intracellular content, including IL-1β and IL-18. **(B)** Non-canonical inflammasome activation is initiated by the detection of cytosolic LPS from gram-negative bacteria by the pro-caspase-11 itself. Activated caspase-11 (caspase-4 or caspase-5 in humans), in turn, induces GSDMD cleavage and consequent pyroptosis.

Pyroptosis is a necrotic form of regulated cell death distinct from necroptosis, mainly due to the requirement of inflammatory caspase-1 and/or caspase-11 (murine caspase-11 corresponds to caspases-4 and -5 in humans) [(136); Figure 3). It is the result of pore formation in the plasma membrane that increases osmotic pressure ensuing in osmotic lysis and, consequently, the release of the intracellular content, including pro-inflammatory cytokines and DAMPs (137). Although distinct from the typical oligonucleosomal fragmentation observed during apoptosis, DNA fragmentation is also a hallmark of pyroptosis, which seems to occur independently of the caspase-activated DNase (CAD) (138).

Pyroptosis is a form of cell death initiated in response to the engagement of certain members of the PRRs, which are capable of assembling complex structures called inflammasomes. These platforms are composed by a sensor protein, either from the NLR or the pyrin and HIN domain-containing protein (PYHIN) families of cytosolic PRRs, in addition to the adaptor molecule apoptosis-associated speck-like protein containing a caspase activating and recruitment domain (ASC) and pro-caspase-1. There are five major types of the so-called canonical inflammasomes—NLR pyrin domain-containing 3 (NLRP3), NLRP1, neuronal apoptosis inhibitory protein (NAIP)/NLR CARD-containing 4 (NLRC4), absent in melanoma 2 (AIM2), and PYRIN inflammasomes (139). Activation of one of these cytosolic sensors in response to PAMPs, DAMPs or cytosolic disturbances such as ionic imbalance leads to the recruitment and activation of caspase-1 either directly or through the ASC adaptor molecule [(140); Figure 3). Besides the induction of pyroptosis, caspase-1 also leads to the processing and release of the pro-inflammatory cytokines IL-1β and IL-18 (139).

In contrast to the canonical inflammasomes, which require multicomplex structures, the non-canonical inflammasome seems to be composed solely by pro-caspase-11, which plays the role of the sensor as well as the executor (141, 142). During intracellular gram-negative bacteria infections, Lipid A, a component of LPS, can directly bind to the CARD domain of pro-caspase-11 (143), which gets activated and induces pyroptosis. Interestingly, the non-canonical caspase-11 inflammasome acts independently of LPS recognition by TLR4 and does not directly induce IL1-β and IL-18 maturation [(141, 142); Figure 3). In monocytes, however, non-canonical inflammasome stimulation may result in minor production of IL-1β and IL-18 through the bystander induction of NLRP3 activation (144). Interestingly, LPS-induced lethal shock is driven by the activation of the non-canonical inflammasome. Since IL-1β and IL-18 release are not a major outcome of caspase-11 activation, the exacerbated inflammatory response observed in sepsis seems to be mainly driven by pyroptosis, probably due to the efflux of DAMPs, such as High Mobility Group Box 1 (HMGB1) and IL-1α [Table 1; (145)].

Such non-canonical inflammasome-mediated responses have drawn the attention of different research groups that became interested in unraveling the relevant as well as the pathogenic caspase-11 downstream targets. In 2015, two concurrent studies reported that Gasdermin D (GSDMD), a member of the GSDM family, was the effector component of the non-canonical inflammasome pathway (146, 147), which was later confirmed by a third study (148). Kayagaki and colleagues performed N-ethyl-N-nitrosourea mutagenesis screening for mutations that compromised LPS-induced IL-1β release and pyroptosis (146) while Shi and colleagues employed the clustered regularly interspaced palindromic repeat (CRISPR)/Cas9 genome-editing screens in TLR4 deficient mouse bone marrow-derived macrophages for guide RNAs that protected from LPS-induced cell death (147). Both studies hit GSDMD as a substrate for caspase-11 and the effector of pyroptosis. GSDMD is composed by a C-terminal and a N-terminal domain linked by a long loop. Caspase-11 cleaves an aspartate residue within the linking loop, releasing the N-terminal fragment from the inhibitory C-terminus (146, 147). The N-terminal domain, also called Pore-Forming Domain (PFD) (149) oligomerizes and associates with lipids in the inner plasma membrane to form 10–33 nm pores leading to cell swelling and eventually to cell lysis (150–153). Importantly, it was also demonstrated that caspase-1 cleaves GSDMD at the same site as caspase-11, establishing that GSDMD is also required for the canonical inflammasome-driven pyroptosis (147).

Until the discovery of GSDMD as the pyroptosis executioner, the physiological function of GSDM proteins was largely unknown. However, recent studies described that the PFD is highly conserved among several members the GSMD family. Indeed, expression of PFD from GSDMA, GSDMA3, GSDMB, GSDMC, GSDME, or GSDMA3 in HEK293 was able to induce pore formation and a cell death phenotype similar to pyroptosis (147, 151). Moreover, GSDMA3 cleavage by caspase-3 in HEK293 and macrophages results in a secondary necrotic cell death after apoptosis (154). This necrotic cell death might contribute to hearing loss in GSDMA3 spontaneous mutations that are associated with deafness (155). Thus, given the cytotoxic activity of different GSDM PFD, some authors have proposed a redefinition of pyroptosis as a GSDM-mediated cell death (146). However, it is controversial how other GSDM members are activated and whether these proteins participate in cell death pathways. Also, GSDMD seems not to be required for pyroptosis during prolonged inflammasome activation in response to the classical agonists, ATP, and flagellin (146). Moreover, in the absence of caspase-1 protease activity, caspase-8 accounts for GSDMD-independent cell death in response to inflammasome agonists (156–158). Since some of these processes share features of pyroptosis, it is hard to define pyroptosis solely as being a process of cell death regulated by inflammatory caspases or mediated by GSDM proteins, since we can find exceptions to the rules that govern both concepts.

From the biological point-of-view, cell death by pyroptosis results in a fast removal of infected cell leading to the elimination of the replication niche. Conversely to the previous idea of liberation of bacteria to the extracellular milieu by pyroptotic cells (159), the current knowledge predicts that, instead, the damaged bacteria remain trapped within the pyroptotic corpses. This structure is called pore-induced trap (PIT) and it prevents bacterial dissemination (160, 161). Despite that PIT does not directly kill intracellular bacteria, pyroptosis renders them more susceptible to H_2_0_2_, to the antimicrobial peptide polymyxin B and to the antibiotic ciprofloxacin (157). As a consequence, the recovered bacteria from PIT are less capable to infect neighbor cells.

The inflammatory milieu created by the release of the intracellular content from pyroptotic cells recruits circulating phagocytes to the infectious site. Subsequently, neutrophils efferocyte the PIT and kill the pathogen by a mechanism dependent on reactive oxygen species (ROS) (161). Extracellular bacteria can also be controlled by the action of antimicrobial peptides (160, 161) and potentially by the GSDMD N-terminal domain released during cell lysis due to its affinity to cardiolipin and phosphatidylserine expressed in some bacterial cell membranes, such as *Escherichia coli* and *Listeria monocytogenes* (152, 162). Interestingly, canonical and non-canonical inflammasomes are required for intestinal epithelial cells (IECs) responses to infections (163, 164). The activation of NLRC4 inflammasomes in IECs results in a lytic cell death prior to a non-conventional process of cell expulsion that contributes to control bacterial replication. Although caspase-1 and Gasdermin-D were required for IEC pyroptosis, both molecules were dispensable for cell expulsion, demonstrating that coordinated inflammasome responses in IECs are important to prevent bacterial translocation to deeper tissues (163, 164).

Interestingly, neutrophils seems to be more resistant to pyroptosis than macrophages in response to *Salmonella* and are able to maintain a sustained IL-1β production and secretion, which could be important to control the infection (165). However, Kambara et al. (166) recently described that a specific neutrophil elastase (ELANE) is able to cleave GSDMD independently of caspases activity, promoting a lytic cell death in these cells. Interestingly, these authors demonstrated that GSDMD-dependent neutrophil death impairs the control of extracellular bacteria *E. coli*, thus suggesting that GSDMD could exert an anti-inflammatory role depending on the infection context.

In addition to its role in the elimination of replicative niche, the pyroptosis machinery is involved in IL-1β and IL-18 release. As these cytokines lack the signal peptide, their release is considered to occur by non-conventional pathways (167). Among the different pathways that have been proposed to explain their secretion, mechanisms involving cell death are particularly subject to intense debate in the literature. Growing evidences suggest that IL-1β can be released by viable monocytes (168), dendritic cells (DCs) (169), and macrophages (170). GSDMD pore is large enough to allow IL-1β release concomitant with the influx of cationic ions (148). Notably, in viable cells, GSDMD seems to be required for IL-1β translocation to the extracellular space in response to stimuli that hyperactivate phagocytes, such as oxidized phospholipids (oxPAPC) in DCs or LPS in human monocytes (170–172). Nonetheless, it is difficult to establish whether the cells were actually viable, since cell death can precede cell lysis, thus suggesting that pyroptosis and cell lysis can be uncoupled events (173). Moreover, the assessment of cell death by the detection of lactate dehydrogenase release (LDH), used in several studies as the only viability assay, might be insufficient to discriminate viable cells from dying cells since both viable and unviable cells can release LDH to the cell culture (170, 173, 174).

Although many studies have demonstrated the requirement of canonical and non-canonical inflammasomes to host defense against pathogens, the precise contribution of pyroptosis and other inflammasome-related mechanisms are poorly understood and arose mainly from *in vitro* assays or bacterial infection models in mice deficient for molecules that compose these platforms (159, 175). In *L. monocytogenes, S. typhimurium*, and *B. thailandensis in vivo* infections, the lack of caspase-1/11 was more deleterious to the host than IL-1β/IL-18 deficiency (176–179), while in mice infected with *F. novicida*, the treatment with recombinant IL-1β/IL-18 only partially recovered the resistance to infection, suggesting that cytokine secretion was not sufficient to protect the host (180). The susceptibility of GSDMD-deficient mice seems to correlate with that demonstrated by caspase-1/11-deficient mice, although the deletion of GSDMD also culminated in reduced IL-1β/IL-18 release (146–148). Even though (a) IL-1β/IL-18 secretion might occur independently of pyroptosis and (b) caspase-1 or caspase-11 deletion is more severe than IL-1β/IL-18 ablation in some bacterial infections, usually cytokine release and cell death are overlapping events necessary for optimal host defense (159). In any case, the susceptibility of GSDMD and other GSDM deficient mouse strains to infectious agents and its comparison to mice lacking caspase-1, caspase-11, IL-1β, and/or IL-18 remains to be established, especially in non-bacterial infection contexts. For example, despite clear evidences of the involvement of inflammatory caspases in the host control of some fungal infections such as *Candida albicans, Aspergillus fumigatus*, and *Paracoccidioides brasiliensis* (172), the requirement of GSDMD to cell death and the consequences to the host resistance against these infections is still to be elucidated.

Notwithstanding, the highly pro inflammatory outcome of pyroptosis as well as the cell loss can be prejudicial to the host during the response to pathogens. In HIV patients, the quiescent CD4 T cells depletion seems to be mainly mediated by pyroptosis (181, 182). During HIV abortive infection, the engagement of the interferon-gamma-inducible-protein 16 (IFI16) in response to cytosolic viral DNA leads to inflammasome assembly and caspase-1 mediated CD4 T cells pyroptosis in lymphoid tissues (181, 182). Interestingly, co-cultivation of lymphoid-derived cells sensitizes blood-derived CD4 T cells to HIV-induced pyroptosis (183). Moreover, pyroptotic peripheral blood CD14^+^CD16^−^ monocytes from HIV-infected patients release ASC specks, a hallmark of inflammasome activation. Therefore, besides the depletion of CD4 T cells, pyroptosis of CD4 T cells and monocytes contributes to the chronic inflammation that characterizes the disease (184).

The identification of the non-canonical inflammasome and the discovery of GSDMD as the executioner of pyroptosis have expanded our understanding of the mechanisms driving this type of cell death. However, further studies are necessary to elucidate the precise role of inflammatory and non-inflammatory caspases and the participation of members from GSDM family and/or other effector proteases in the molecular regulation of pyroptosis. In addition, the understanding of its role during infection or inflammatory processes *in vivo* will contribute to better understand the biological relevance of this regulated cell death induced in response to the PRRs activation.

## PRR sensing of cell death and cell death products

The notion that cells undergoing cell death release or expose several intracellular molecules regardless of the accidental nature or the different regulated death programs (apoptosis, necrosis or pyroptosis) is widely recognized. Although mainly non-inflammatory in the intracellular space, molecules released/exposed from damaged cells can participate in the activation of inflammation and immune responses. Indeed, a broad range of receptors, including PRR, sense these DAMPs and alert the immune system by inducing immune cell migration, increasing phagocytosis by macrophages and DCs, stimulating the production of pro-inflammatory cytokines or even contributing to the maturation of DCs, among other key functions (72, 80, 185). A number of studies have been dedicated to the characterization of putative DAMPs, and it became apparent that the type of cell death, as well as the nature of cell death stimuli, influence the quality and quantity of DAMPs release (Table 1).

Importantly, the stress or damage before the cellular demise itself is determinant to set in motion a sequence of events leading to an immunogenic cell death (ICD). The sensing of this stress regulates the cell death process thus initiating signaling pathways that will actively—or not—generate danger signals (186). Other DAMPs will be passively released as a result of membrane rupture during necroptosis or pyroptosis. These DAMPs define in part the immunogenicity of cell death, but are not sufficient to elicit a specific anti-tumor immune response, for instance. Indeed, they are released or exposed by the dying cells and act as adjuvant providing that antigens are exhibited conjointly (187). In contrast, a non-immunogenic cell death does not provide the required levels of DAMPs and antigens to evoke an adaptive immune response (187).

Together, these concepts redefined the widely accepted paradigm stating that apoptosis is always a silent cell death modality as opposed to necrosis, which is inflammatory and immunogenic. Therefore, a non-immunogenic apoptosis is characterized by the absence of plasma membrane leakage and the rapid phagocytosis of apoptotic bodies prevents the release of DAMPs and the consequent inflammatory reaction. Indeed the apoptotic process reduce cell immunogenicity by diverse ways including, (1) preservation of intracellular structures and plasma membrane, thereby blocking the release of DAMPS; (2) reduction of cellular volume, by condensation of the nucleus and shedding of small vesicles, which favors its rapid elimination by the surrounding tissue; (3) expression of “find-me” and “eat-me” signals, which increases the speed of cell clearance (12, 188); (4) inhibition of the production of interferons and pro-inflammatory cytokines, as the DNA is being chopped and condensed (189); (5) induction of an antigenic tolerance in the engulfing APCs (190); and (6) post-translational modifications on DAMPS and alarmins that decrease their pro-inflammatory potential (191, 192).

Interestingly, depending on the trigger, apoptosis can be immunogenic. Indeed, some chemotherapeutic agents, such as anthracyclines, as well as radiation and hypericin-based photodynamic therapy, were found to strongly prime immune responses through the induction of ICD (65, 185). Among these, immunogenic chemotherapies are well characterized and involve the emission of a number of danger signals. The pre-apoptotic release or exposure on the plasma membrane of ER-chaperones, such as calreticulin and Heat Shock Proteins (HSPs), constitutes an early event of ICD, which relies on the induction of an ER-stress. Calreticulin promotes the uptake of dying cells by DCs (72) and the inhibition of its exposure during anthracycline-induced apoptosis of murine tumor cell lines abolished their immunogenic potential (72). The early apoptotic secretion of ATP, which binds to P2X7 or P2Y2 purinergic receptor on DCs, stimulates the formation of the NLRP3 inflammasome, thereby inducing the release of IL-1β (66). Moreover, ATP released by dying cells undergoing ICD is responsible for the recruitment and differentiation of myeloid precursors into inflammatory DCs, mediating a specific antitumor immune response (193). Passive release of the nuclear protein HMGB1 occurs during secondary necrosis (i.e., late-stage apoptotic cells), which interacts with TLR4 on DCs, and through Myd88 signaling, enables efficient tumor antigen processing and cross-presentation (80). Additionally, anthracyclines have been shown to induce the release of RNA, thereby stimulating TLR3 as a mimic of viral infection. Activation of TLR3 is then responsible for type I IFNs production that acts in an autocrine and paracrine manner to promote the secretion of CXCL10 (194). Release of Annexin A1 has also been described after anthracyclines treatment, stimulating the Formyl Peptide Receptor 1 (FPR1), thus directing the final trajectory of DCs to dying tumor cells (195).

Besides chemotherapeutic agents, bacterial and viral infection can also trigger an immunogenic apoptosis. In this case, PAMPs, such as LPS or double-stranded RNA, expressed by the pathogen can stimulate TLR signaling and induce an immune response. Indeed, phagocytosis of infected apoptotic cells could trigger TLR activation and lead to IL-6 and TGF-β production that induce the development of infection-specific as well as self-reactive T_H_17 cells, linking infection to autoimmunity (196, 197). Finally, defects in mechanisms of apoptotic cell clearance are linked to autoimmunity disorders, including lupus and rheumatoid arthritis, likely due to the increased risk of loss of cell integrity with the consequent release of DAMPs and increased availability of circulating self-antigens (198, 199).

Accidental or programmed forms of necrosis are responsible for the release of an usually larger panel of DAMPs compared to apoptotic cells, mainly due to plasma membrane permeabilization. Also, RIPK1-mediated activation of the NF-κB pathway, through upregulation of pro-inflammatory cytokines and increased antigen presentation, was reported to play a role in the immunogenicity of necroptotic cell death mode (186). Recently, it was show that necroptosis is accompanied by the release of the classical and potent DAMPs—HSPs, ATP, and HMGB1 (200, 201). While HSPs were shown to stimulate TLR2 and 4, inducing DCs maturation, HMGB1 was reported to interact with TLR3, 4 and 9, as well as RAGE, to activate DCs and macrophages (202–204). Necrotic cells can potentially release intact mitochondria, a major source of ATP, which may activate the NLRP3 inflammasome resulting in IL-1β secretion and neutrophil recruitment (67, 96, 205). Moreover, mitochondrial DAMPs, such as formyl peptides and mitochondrial DNA, can potentially act on FPR1 and TLR9, respectively, inducing neutrophils recruitment and degranulation (115, 97). Additionally, Mincle, the C-type lectin receptor 4E was reported to interact with the necrotic DAMP SAP130 (spliceosome-associated protein 130), normally involved in spliceosomes assembly. The stimulation of this PRR was also reported to induce recruitment of neutrophils (106). Uric acid has been described as a product of accidental necrosis (108). Once released, this DAMP can precipitate and form monosodium urate (MSU) crystals able to induce the expression of costimulatory molecules by DCs, as well as activating the NLRP3 inflammasome to trigger the production of IL-1β and IL-18 (206, 207). RNA and double-stranded DNA (dsDNA) are also released during necrotic cell death, and while RNA stimulates TLR3, dsDNA stimulates TLR9 and two members of the RLR family, RIG-I and MDA5, responsible for the release of IFN-β and CXCL10 through IRF3 and NF-κb pathways. The AIM2 inflammasome can sense dsDNA released by necrotic cells, thereby inducing IL-1β secretion (204). Finally, it is important to mention that some proteins considered DAMPs also stimulate receptors that are not PRRs. For instance, IL-1α acts on IL-1R1 to trigger an inflammatory response mediated by the Myd88 pathway (208) and full-length IL-33 is another DAMP released during necrosis, with immunological property due to the absence of caspase processing (191).

## Conclusions

**Figure 4:**
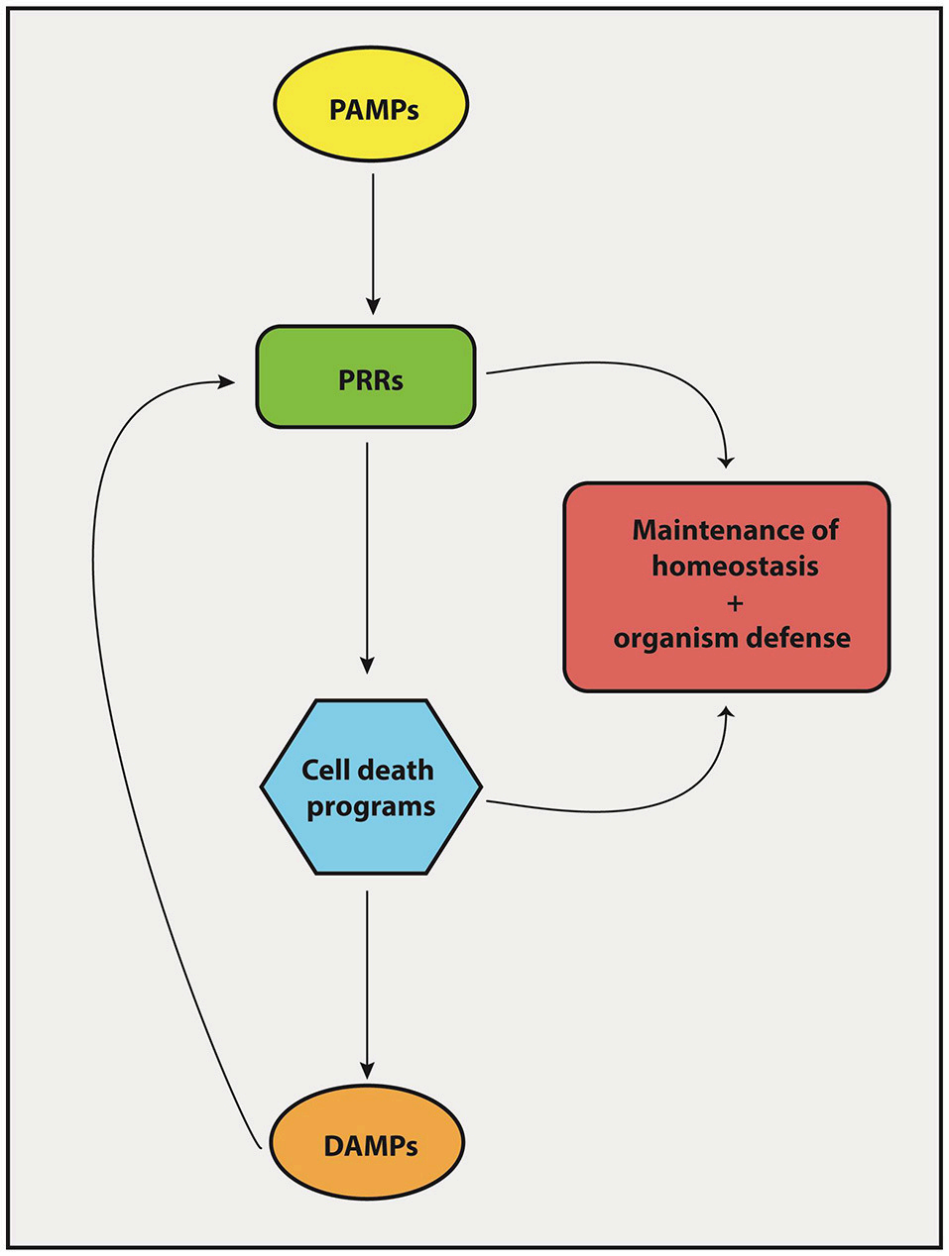
Interplay between PRRs and cell death mechanisms. The engagement of PRRs in response to PAMPs induces the activation of different cell death machineries in order to promote tissue homeostasis and host-defense against pathogens. Importantly, cell death products known collectively as DAMPs forms a feedback loop that stimulate PRRs to induce inflammatory/immune responses.

The protective response of our body against pathogens and tumor cells depends on proper activation of both innate and adaptive immunity. Particularly, macrophages and DCs reside on the center of these two arms of immunity. They are powerful antigen-presenting cells that may elicit effector T cell responses (protection) or induce T cells to become regulatory (tolerance), depending on their activation status. They express PRRs, which are very ancient proteins that help us identify and react to pathogens and danger signals. Upon engagement, through the interaction with conserved molecular patterns frequently associated with pathogens (PAMPs), PRRs trigger a series of biochemical signaling cascades that activates pro-inflammatory programs on DCs that enable the differentiation of antigen-specific T cells into protective effector TH1, TH2, and TH17 cells. PRR engagement also triggers programs of cell death, particularly necroptosis and pyroptosis, the necrotic forms of cell death associated with a pro-inflammatory outcome (Table 2). These forms of cell death release larger amounts of DAMPs, which in turn, stimulate surrounding cells via PRRs, thus constituting a positive feedback loop capable of amplifying host defense mechanisms (Figure 4). Apoptosis, on the other hand, is a cell death program mostly related to non-inflammatory outcomes and likely to take major role in the maintenance of homeostasis by “silently” eliminating unwanted or damaged cells. However, apoptosis may also participate in elimination of infectious agents or tumor cells. Therefore, recognition of pathogen- and damage/danger-associated molecules by the same set of immune receptors (PRRs) is a powerful strategy that bridges intrinsic cell death programs and complex immune cell interactions to preserve homeostasis and at the same time protects the organism against infection and cellular transformation (Figure 4).

**Table 2:** PRR agonists and consequent cell death program.

**Stimuli/DAMPS**	**PRRs**	**Cell death mode**	**References**
Pam3CSK4	TLR1	Apoptosis	(209, 210)
Pam3CysK Lipoproteins	TLR2	Necroptosis Apoptosis	(27, 211, 212)
Poly(I:C)	TLR3	Necroptosis Apoptosis	(211, 213, 214)
LPS HMGB1	TLR4	Necroptosis Apoptosis	(211–213, 215, 216)
Flagellin	TLR5	Necroptosis Pyroptosis	(211)
CpG DNA	TLR9	Necroptosis	(211)
LPS	CASPASE-11	Pyroptosis	(141–143)
Crystals/particulate-matter	NLRP3	Pyroptosis	(207, 217–220)
ATP	NLRP3	Pyroptosis	(221)
Bacterial pore-forming toxins	NLRP3	Pyroptosis	(221–224)
Bacterial RNA	NLRP3	Pyroptosis	(225)
dsRNA	NLRP3	Pyroptosis	(226)
Saturated-fatty acids	NLRP3	Pyroptosis	(227)
Flagellin T3SS/T4SS needle and inner rod proteins	NAIP/NLRC4	Pyroptosis	(228–233)
dsDNA	AIM2	Pyroptosis	(234)
*Bacillus anthracis* protective agent	NALP1	Pyroptosis	(235)
Muramyl dipeptide	NALP1	Pyroptosis	(236)
Toxin-modified RHO GTPase	PYRIN	Pyroptosis	(237)
ATP	P2X7	Apoptosis	(238, 239)
ssRNA shRNA	RIG-I	Necroptosis Apoptosis	(57, 240)
dsDNA Genomic RNA	DAI (DLM-1/ZBP)	Necroptosis Apoptosis	(56, 241)

*AIM2, absent in melanoma 2; DAI (DLM-1/ZBP), DNA-dependent activator of IFN-regulatory factors; dsDNA, double stranded DNA; dsRNA, double stranded RNA; HMGB1, high-mobility group box 1 protein; LPS, Lipopolysaccharides; NAIP/NLRC4, NLR family CARD domain-containing protein 4; NALP1, NACHT, LRR and PYD domains-containing protein 1; P2X7, P2X purinoceptor 7; PAM3CSK4/Pam3CysK, TLR2 receptor agonist; Poly(I:C), Polyinosinic:polycytidylic acid; RIG-I, retinoic acid-inducible gene-I-like receptors; shRNA, short hairpin RNA; ssRNA, single stranded RNA; T3SS/T4SS, Type III/IV secretion system; TLR, Toll-like receptor*.

## Author contributions

LM designed the figures. LZ and SA prepared Table. GA-M, RW, and KB designed the review organization. All authors contributed to the writing, reviewed, and approved the manuscript.

### Conflict of interest statement

The authors declare that the research was conducted in the absence of any commercial or financial relationships that could be construed as a potential conflict of interest. The reviewer FD and handling Editor declared their shared affiliation at the time of review.
